# The trilateral interactions between mammalian target of rapamycin (mTOR) signaling, the circadian clock, and psychiatric disorders: an emerging model

**DOI:** 10.1038/s41398-022-02120-8

**Published:** 2022-08-31

**Authors:** Rubal Singla, Abhishek Mishra, Ruifeng Cao

**Affiliations:** 1 Department of Biomedical Sciences, University of Minnesota Medical School, Duluth, MN 55812 USA; 2 Department of Neuroscience, University of Minnesota Medical School, Minneapolis, MN 55455 USA

**Keywords:** Psychiatric disorders, Physiology

## Abstract

Circadian (~24 h) rhythms in physiology and behavior are evolutionarily conserved and found in almost all living organisms. The rhythms are endogenously driven by daily oscillatory activities of so-called “clock genes/proteins”, which are widely distributed throughout the mammalian brain. Mammalian (mechanistic) target of rapamycin (mTOR) signaling is a fundamental intracellular signal transduction cascade that controls important neuronal processes including neurodevelopment, synaptic plasticity, metabolism, and aging. Dysregulation of the mTOR pathway is associated with psychiatric disorders including autism spectrum disorders (ASD) and mood disorders (MD), in which patients often exhibit disrupted daily physiological rhythms and abnormal circadian gene expression in the brain. Recent work has found that the activities of mTOR signaling are temporally controlled by the circadian clock and exhibit robust circadian oscillations in multiple systems. In the meantime, mTOR signaling regulates fundamental properties of the central and peripheral circadian clocks, including period length, entrainment, and synchronization. Whereas the underlying mechanisms remain to be fully elucidated, increasing clinical and preclinical evidence support significant crosstalk between mTOR signaling, the circadian clock, and psychiatric disorders. Here, we review recent progress in understanding the trilateral interactions and propose an “interaction triangle” model between mTOR signaling, the circadian clock, and psychiatric disorders (focusing on ASD and MD).

## General introduction

Circadian rhythms are the approximately 24-h biological rhythms that are found in almost all living organisms on the planet [1]. These rhythms are endogenously driven by circadian clocks, which can function autonomously but are constantly regulated by environmental signals including light, food availability, etc. [2]. Circadian clocks synchronize numerous internal biological processes with the cyclical changes in the external environment, so that organisms can predict and prepare for upcoming environmental changes and adjust their physiological states accordingly [3]. In cells, the circadian clock is driven by interlocking feedback loops of gene expression. Clock genes are widely expressed in almost all cells of the body. In the brain, a variety of neural processes are orchestrated by circadian clocks throughout life. During neurodevelopment, the circadian clock regulates neurogenesis, migration, and progenitor cell differentiation [4, 5]. In the adult brain, circadian rhythms regulate neuronal excitability, synaptic plasticity, learning and memory, mood, and social behaviors [6–8]. Disruption of circadian rhythms can cause mental health problems. In the short term, circadian disruption can cause jet lag-like symptoms, including fatigue, insomnia, difficulty concentrating, etc. [9]. Chronic disruption of circadian rhythms and sleep-wakefulness cycles is often associated with neurological and psychiatric disorders, such as Alzheimer’s disease (AD), Parkinson’s disease (PD), mood disorders(MD), and autism spectrum disorders (ASD), although it is not entirely clear whether circadian dysfunction underlies the pathogenesis of these brain disorders or it is simply a consequence caused by the primary pathophysiological changes in these diseases [10]. There is evidence from neurological and psychiatric disorders where sleep and circadian dysfunction contribute to both the vulnerability to and the development and progress of certain disorders.

**Fig. 1:**
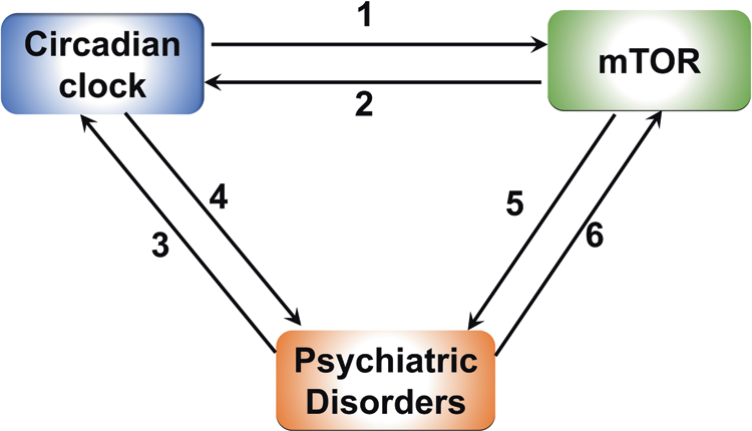
A proposed “interaction triangle” model between the circadian clock, mTOR signaling, and psychiatric diseases. 1. The circadian clock temporally regulates mTOR activities in different tissues. 2. mTOR signaling regulates the central and peripheral circadian clock functions. 3. Psychiatric disorders can cause disruption of circadian rhythms. 4. Circadian dysfunction can contribute to the pathogenesis of psychiatric disorders. 5. Aberrant brain mTOR activities lead to psychiatric diseases. 6. Psychiatric disorders can lead to dysregulation of mTOR activities in the brain.

The mechanistic/mammalian target of rapamycin (mTOR) signaling is an evolutionarily conserved intracellular signal transduction cascade that regulates fundamental cellular processes including cell growth, metabolism, proliferation, and aging from yeasts to humans [11–13]. Dysregulation of mTOR signaling is implicated in a number of common human diseases, including cancer, metabolic syndrome, cardiovascular diseases, and neurological and psychiatric disorders [14]. Pharmacological mTOR inhibitors are clinically applied for immunosuppressing after organ transplantation, cancer chemotherapy, as well as treating some neurological and psychiatric disorders in clinical trials. Recent work has uncovered significant crosstalk between mTOR signaling and the circadian clock in different tissues and systems, suggesting it could be a fundamental mechanism of homeostatic integration between cell metabolism and circadian timekeeping [15]. Along these lines, the activities of mTOR signaling are regulated by the circadian clock and exhibit robust diurnal oscillations in the brain and peripheral systems such as the liver [16, 17]. On the other hand, the fundamental properties of the circadian clock (entrainment, synchronization, speed) are regulated by the mTOR signaling [18–20]. Importantly, mTOR activities are often found dysregulated in brain diseases where the daily rhythms in patients are disrupted. Understanding the crosstalk mechanisms between mTOR and the clock may help to gain new insights into the pathogenic mechanisms of psychiatric disorders and develop novel therapeutic strategies to treat these diseases. Increasing evidence support an “interaction triangle” model between the circadian clock, mTOR signaling, and psychiatric diseases (Fig. 1). In this review, we will first introduce the mammalian circadian system and a role for mTOR signaling in the mammalian circadian clock, and then we will discuss recent progress on our understanding of circadian and mTOR dysfunctions in highly prevalent psychiatric disorders, ASD and MD.

## The mammalian circadian system

In mammals, the circadian system is organized in a hierarchical manner. The central clock is located in the suprachiasmatic nucleus (SCN) of the hypothalamus [21]. The pair of tear drop-like structures is composed of only ~20,000 neurons, but it is essential for circadian timekeeping in animals [22, 23]. The SCN predominantly determines the speed of activity rhythms in animals and serves as the primary responder to the most important clock resetting signal-ambient light [24]. The important functions of the SCN are enabled by its unique anatomical structure. The SCN is located just above the optic chiasm and its ventral neurons receive photic signal via the monosynaptic input from the melanopsin-expressing intrinsically photosensitive retinal ganglion cells via the retinohypothalamic tract [25–28]. As part of the hypothalamus, the SCN is embedded in a cluster of hypothalamic nuclei (e.g., paraventricular nucleus) that play pivotal roles in neural and hormonal control of homeostasis. The rhythmic output from the SCN to these nuclei regulates daily rhythms in the endocrine system and the autonomic nervous system [29–31].

The SCN neurons are highly heterogeneous in their neuropeptide expression, electrophysiological properties, and photic response [32, 33]. The ventral SCN neurons express vasoactive intestinal peptide (VIP) and gastrin-releasing peptide (GRP) and the dorsal SCN neurons express arginine vasopressin (AVP). Cellular oscillators in the SCN are coupled to form a coherent and stable oscillating network. Intercellular synchronization between SCN neurons renders robustness and accuracy to the SCN-generated rhythms [34]. The unique coupling mechanisms distinguish SCN from peripheral circadian oscillators, where the coupling between cellular oscillators is considered weak [34–36]. Vasoactive intestinal peptide (VIP) and GABAergic signaling are important for coupling cellular oscillators in the SCN [37–43]. AVP receptors are essential for the communications between the ventral and dorsal SCN [44]. AVP positive neurons exhibit stronger circadian rhythms in firing rates and membrane properties compared with AVP negative neurons, indicating their important role in transducing SCN rhythms to other brain areas [45]. Light-induced immediate early gene expression and activation of protein kinases first appear in the “core” SCN, including VIP and GRP expressing cells, indicating that the ventral SCN neurons first respond to light stimulation [46–48]. The dorsal SCN neurons in turn received resetting signal from the ventral SCN neurons. Besides SCN neurons, recent studies have uncovered a modulatory role for astrocytes in the SCN [49]. Interestingly. astrocytic clocks alone can drive molecular oscillations in SCN neurons via glutamatergic signals.

**Fig. 2:**
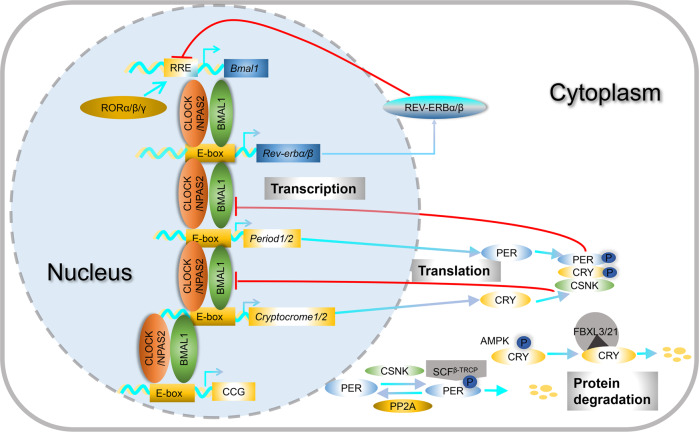
The molecular mechanisms of mammalian circadian timekeeping through the autoregulatory transcriptional-translational feedback loops. The transcription factors CLOCK (or NPAS2) and BMAL1 form heterodimers, which bind to the *cis*-acting element E-box and activate the expression of *Period1/2* and *Crypotochorme1/2*. PER and CRY proteins form multiprotein complexes in the cytoplasm. Once accumulating to a certain level, the PER/CRY complexes translocate into the nucleus, interact with the CLOCK: BMAL1 complex, and repress their own gene transcription. The CLOCK: BMAL1 complex also promotes the transcription of *Rev-erbα/β*. REV-ERBs inhibit the *Bmal1* transcription whereas RORs promote *Bmal1* transcription. PER protein abundance is controlled at the mRNA translation level via an elF4E-dependent mechanism. CRY is phosphorylated by AMPK and PER by CSNK. The levels of CRY and PER are also regulated by phosphorylation and ubiquitin-medicated protein degradation at the post-translational levels. The CLOCK: BMAL1 complex also regulates numerous clock-controlled genes via the E-box enhancer.

Whereas SCN is the master pacemaker, clock genes are widely expressed in almost all cells throughout the body. Clock gene oscillations have been identified in a variety of brain regions [50]. The local brain clocks regulate neuronal properties of individual brain regions and presumably also regulate the brain functions in a time-of-day dependent manner [7]. Phase differences between region-specific clock oscillators are observed, but the coupling mechanisms between these oscillators remain to be fully understood [51]. At the cellular level, the circadian clock is driven by transcriptional/translational genetic feedback loops (TTFL) [52]. Decades of research have identified the framework of feedback mechanisms in different specifies. In mammals, the transcriptional activators BMAL1 and CLOCK (or NPAS2) form a heterodimeric complex to activate E-box-mediated transcription of *Period* (*Per1, 2, 3*) and *Cryptochrome* (*Cry1, 2*) genes [53, 54]. PER and CRY form protein complexes in the cytosol and translocate to the nucleus upon reaching a level enough to suppress their own gene transcription [2, 55]. In a second negative feedback loop, the transcription of *Rev-erb α, β* is promoted by CLOCK: BMAL1. In turn REV-ERBs repress *Bmal1* transcription via the retinoic acid response element (RRE) [56]. The nuclear hormone receptors RORα, β, γ compete with REV-ERBs to activate *Bmal1* transcription [57, 58]. Rhythmic expression of clock output genes (so called “Clock-controlled genes”) is transcriptionally regulated by CLOCK: BMAL1 acting on E-box elements in their regulatory regions [59]. Thus, CLOCK: BMAL1 is not only critical for sustaining the TTFL, but also serves as an important mechanism of clock output. Besides transcriptional mechanisms, the clock gene expression, and levels of clock proteins are also regulated at the levels of mRNA translation and post-translational protein degradation [18, 60–64]. Together, gene expression regulation at different levels ensures the precision and robustness of circadian gene expression (Fig. 2). To be in sync with external and internal environmental changes, the circadian clock must be regulated by extracellular and intracellular signals [3, 65]. As signal transduction process is complex, our understanding of the key signal transduction events that couple extracellular and intracellular signals to clock gene expression is not complete. In particular, the crosstalk mechanisms between cell metabolism and core clock mechanisms remain to be fully understood.

## mTOR signaling and the circadian clock

**Fig. 3:**
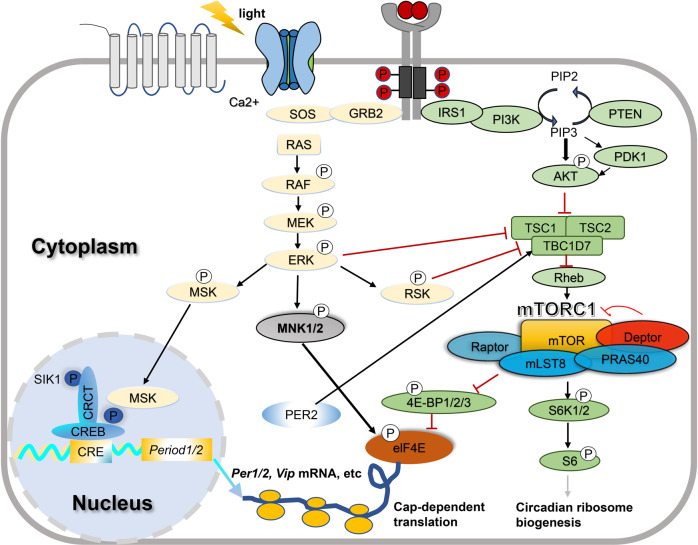
The interaction between the circadian clock and mTOR-related signaling transduction pathways. Light at night activates ERK MAPK and mTORC1 pathways in the SCN by the neurotransmitter glutamate and PACAP. ERK MAPK in turn activates MSK to phosphorylate CREB and activated *Per* transcription. ERK MAPK also activated MNK, which in turn phosphorylates the cap-binding protein eIF4E at Ser209 and regulate mRNA translation. The mTORC1 activation also regulates translation through downstream translation pathway effectors such as S6Ks and 4E-BPs, etc. Phosphorylated S6K regulates circadian ribosomal biogenesis. Phosphorylation of 4E-BP activated eIF4E dependent translation. The ERK MAPK and mTOR pathways converge on eIF4E to regulate cap-dependent translation of *Per1*, *Per2*, *Vip* mRNAs, which play important roles in photic entrainment of the circadian clock and SCN cell synchronization. Circadian mTOR activities are controlled by the circadian clock via complex mechanisms, one of which may be through the interaction of Per2 with TSC1.

Protein synthesis (mRNA translation) is the most energy-consuming step in gene expression and subject to delicate regulation [66]. mTOR is a highly conserved Ser/Thr protein kinase that forms a complex signaling network and integrates extracellular and intracellular signals to impinge on machineries of mRNA translation and control protein synthesis [11–13]. mTOR forms two functionally distinct protein complexes mTORC (mTOR complex) 1 and mTORC2 (Fig. 3). Recent work has found it regulates various neuronal processes including neural progenitor cell growth and differentiation, synaptic plasticity, learning and memory, hormone secretion, food uptake, and sleep [67–72].

mTORC1 activity has been extensively studied in the SCN [16, 18, 19, 47, 73]. mTORC1 activity is highly rhythmic in the SCN, high during the day and low at night. It is also highly responsive to light stimulation at night [16]. Transient light exposure can induce rapid (within 15 min) upregulation of the S6K1 and 4E-BP phosphorylation in the ventral SCN [73]. Blocking mTORC1 activation in the SCN before light can significantly affect light-induced behavioral phase shifts in mice [47]. In mice without 4E-BP1, clock re-entrainment by a shifted light/dark cycle is much more quickly, as compared to the wild-type mice. Mechanistic studies found enhanced translation of the precursor protein of VIP, prepro-VIP in the brain of *Eif4ebp1*KO mice, indicating that enhanced VIP signaling may underlie the circadian behavioral changes in *Eif4ebp1* KO mice [18]. Genetic repression of mTOR signaling only in VIP neurons can lead to cellular and behavioral changes similar to those seen in the VIP mutants [19].

Beyond the SCN central clock, mTOR rhythms have been found in numerous cells and tissues and mTOR regulates fundamental functions of the peripheral circadian clocks [15]. It is found that mTOR signaling controls the circadian clock properties in a variety of tissues and organisms, which suggests mTOR is a conserved circadian regulator [15, 20, 74, 75]. Strong circadian rhythms of mTOR activities are found in different tissues and cells in mammals [15]. Pharmacological mTORC1 inhibitors can dampen circadian rhythms of PER2 rhythms in hepatocytes, liver slices and SCN slices in a reversible manner [20]. Genetic mTOR repression can dampen the amplitude and lengthen the circadian period in cellular oscillators, SCN slices, and whole animals [20]. mTOR pharmacological inhibitors can markedly slow down and damp the rhythms in the liver clock. Conversely, mTOR activation can accelerate the speed of cellular clocks [20]. In addition, the mTORC1 target S6K1 was found to phosphorylate the clock protein BMAL1 and regulate rhythmic translation [76]. In TSC mutants where mTOR activities are constitutively elevated, mice demonstrate a shorter wheel-running period and disrupted core body temperature rhythms in constant conditions [77].

How the circadian clock regulates mTOR activities is not completely clear. One study found that the circadian protein Period2 recruits TSC1 to the mTORC1 complex and suppresses mTORC1 activity [78]. Specific functions of the ribosomal protein S6 kinases (S6Ks) and mTORC2 in the circadian clock remain to be identified. There is an urgent need to fill in these knowledge gaps because mTOR inhibitors (rapalogs) are FDA-approved drugs and are being widely used to human patients without regard to the potential effects of these therapeutics on the rhythmicity of gene expression. Knowledge on physiological functions of mTOR would also be important to understand the mTOR-dependent mechanisms whereby circadian dysfunction is involved in the pathogenesis of brain diseases.

## Circadian dysfunction, mTOR, and ASD

### Introduction of ASD

ASDs are an array of neurodevelopmental disorders that are characterized by core behavioral symptoms, including deficits in social interaction and/or communication, repetitive behaviors, and restricted interests. ASD starts in early childhood around the age of 3 years and persists throughout life [79]. People with ASD commonly experience other comorbidities such as memory and learning deficits, seizures, motor incoordination, changes in sensory perception, anxiety, and sleep disturbances [80–82]. According to the data released by the Centers for Disease Control and Prevention (CDC) and Autism and Developmental Disabilities Monitoring network, about 1 in 44 (2.3%) children in the United States have been identified with autism in 2018 [83]. Also, it is four times more common in boys than girls [84]. The etiological factors of ASD remain to be fully understood. The environmental and genetic causes of this disorder are often studied separately, however, ASD may be caused by a combination of both factors. There are no medications that treat the core symptoms of ASD but a combination of multiple treatments (behavioral, social, educational, medical, etc.) are used to reduce symptoms that interfere with daily functioning and quality of life.

Despite the high prevalence of ASD worldwide, the pathogenic mechanisms are not fully understood, although a number of ASD risk genes have been identified. The pathological changes in the brain of ASD patients are not consistent and can comprise alterations in the cell size, synaptic growth as well plasticity, changes in the morphology of the dendritic spines [85, 86]. Abnormalities in the neurotransmission has been implicated in the development of ASD, ranging from an imbalance in glutamatergic and GABAergic (excitatory/inhibitory) neurotransmitters as most explored to dopaminergic, adrenergic, serotonergic, and endo-cannabinoid systems among the less explored pathways [87–89]. At the cellular and molecular level, changes include altered neural circuits as well as synaptic plasticity, changes in the morphology of the dendritic spines, abnormal levels of synaptic proteins, and impaired synaptic homeostasis [90]. Due to the complexity of the symptoms, understanding the exact pathophysiology of ASD remains a formidable challenge. This in turn hampers the development of new drug strategies to treat this disorder. Indeed, until today the antipsychotics risperidone and aripiprazole are the only FDA-approved drugs for the management of ASD [91, 92]. Nevertheless, the clinical use of these drugs in ASD is limited as they lack the ability to treat the core ASD symptoms and are accompanied by various side effects [93–95]. Therefore, there is an urgent need to elucidate the mechanistic alterations responsible for ASD.

### Circadian clock and sleep problems in ASD

Circadian dysfunctions are frequent comorbidities of ASD [96]. Studies suggest that abnormalities in the cortisol, melatonin levels, as well as disrupted sleep wake cycle, have been implicated as underlying features of ASD [97]. The circadian clock controls diurnal oscillations of cortisol and melatonin levels and the timing of sleep onset. The alterations in the circadian timing system may be associated to various neurobehavioral changes including sleep problems, behavioral and cognitive alterations [98]. Literature suggests that ~50–83% ASD individuals show sleep problems, comparing to <30% in the normal population [99, 100]. The most frequently reported circadian and sleep problems in ASD include the symptoms of insomnia indicated by inability to sleep or stay sleep and circadian rhythm sleep wake disorders manifested as a misalignment in the endogenous circadian rhythms and the external environment [101]. In particular, the sleep dysregulations and changes in the biological rhythms lead to a worsening of behaviors, decreased seizure threshold, sensory abnormalities, and an overall affected quality of life in these ASD children [102]. Also, a decrease in the levels of melatonin as well as decreased melatonin synthesis was repeatedly reported in ASD individuals [97, 103, 104]. The therapeutic benefits of melatonin treatment in ASD patients range from improving the sleep latency and sleep quality to improving the behavioral impairements [105–107]. A randomized clinical trial found aglomatine, an analogue of melatonin to be effective in the treatment of insomnia and sleep problems in ASD patients [108]. All these data suggest the relevance of an underlying impairment of the circadian timing system to the behavioral ASD symptoms. Therefore, studying the mechanisms associated to circadian dysregulation in ASD may be helpful in identifying the early biomarkers for improving the diagnosis and lifelong prognosis of ASD.

The circadian system is complex, as almost all cells in the body exhibit circadian rhythms of gene expression. As aforementioned, the cellular circadian rhythms are generated by auto regulatory feedback loops of clock genes. Accumulating evidence supports the association between clock gene variants and ASD [109]. Nicholas et al. screened SNPs in eleven clock and clock related genes in over 100 ASD children and their parents and found a significant allelic association of two circadian system related genes (*PER1* and *NPAS2*) with two SNPs in each gene [110]. In addition, a high proportion of all possible haplotypes in *NPAS2* were also significant in ASD individuals [110]. Thus, this study supported the hypothesis that the epistatic clock genes may be involved in the ASD etiology. A genome-wide study by Hu et al. reported altered expression of various circadian genes in the lymphoblastic cell lines from ASD individuals as compared to their respective controls [111]. Clock gene sequencing studies identified SNPs in multiple clock genes (*TIMELESS, NR1D1, RORA, ARNTL2, PER1, PER2, PER3, BMAL1, CLOCK*) in ASD patients [109, 112–116]. The functional mutations of *NR1D1* were also detected in ASD subjects with and without sleep disorders [112]. A study by Hoang et al. suggested a possible role of *PER2* gene in the sleep dysregulation of ASD individuals [117]. Whole exome sequencing studies identified de novo loss-of-function variants in *PER2*, *RORB* and *CSNK1E* [118–121]. These studies suggest that the functional deficits of various circadian relevant genes may be related to ASD etiology and pathophysiology.

Next, increasing studies have found circadian clock dysfunctions in animal models of ASD. For example, a recent study by Delorme et al. showed changes in the circadian rhythms indicated by altered locomotor activity rhythms in the mouse model of maternal immune activation of ASD [122]. In particular, the mice whose mothers were injected with Poly I:C in pregnancy developed increased subjective day activities, which suggests a link between circadian rhythm and ASD [122]. *SHANK3* mutations are involved in ASD pathogenesis. Interestingly, the protein levels of synaptic *SHANK3* exhibit circadian rhythms in the mouse hippocampus [123]. A study shows that the *Shank3*^*+/-*^mouse, a genetic mouse model of ASD, exhibits altered light sensitivity in the SCN clock [124]. Along these lines, another study on mice with a deletion in *Shank3* exon 21 reports that these mice have problems falling asleep and exhibit transcriptional down-regulation of clock gene *Per3* and *Nr1d1* in the prefrontal cortex [125]. These mice also exhibit impaired circadian wheel-running activities in constant darkness [125]. *SCN2A* is another important ASD risk gene. Ma et al. found that the *Scn2a* deficient mouse, an ASD genetic animal model, exhibits increased wakefulness and reduced non-rapid-eye-movement sleep. *Scn2a* deficiency also disrupts the spontaneous firing pattern in SCN neurons [126]. Vijaya Shankara et al. found significant changes in circadian wheel-running behavior in the BTBR T^+^Itpr3^tf^/J (BTBR) mouse, an idiopathic model of ASD [127]. The BTBR mice exhibit shorter free running period and higher level of activities as compared to WT C57BL/6J mice. These mice also show increased clock resetting in response to light stimulation at night and accelerated clock resetting to advanced and delayed light cycles. A study by Ferraro et al. demonstrated that in the valproic acid-induced model of ASD, the rhythmic Bmal1 expression in the SCN and diurnal rhythms of corticosteroid are disrupted in a sexually dimorphic manner [128]. Animals exhibit impaired circadian wheel-running behavior under constant dark conditions and reduced clock resetting in response to light at night [128]. Together, these animal studies demonstrate that circadian functions and clock gene expression are often disrupted in ASD animal models.

**Fig. 4:**
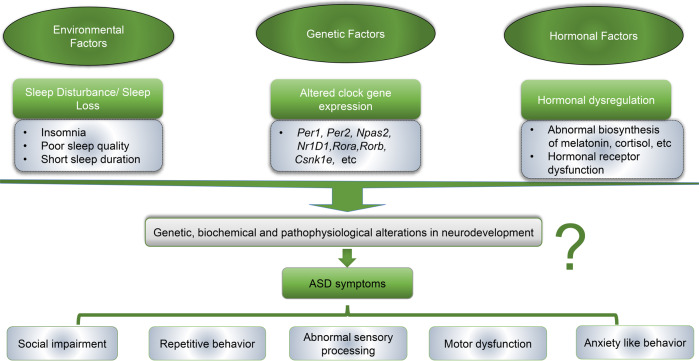
A hypothetical model whereby circadian and sleep dysfunctions might contribute to the pathogenesis of autism spectrum disorders. The environmental, genetic and hormonal risk factors cause sleep and circadian disturbances, which may in turn lead to impairments in neurodevelopment and cause neurodevelopmental disorders such as ASD.

Emerging evidence supports the hypothesis that circadian dysfunction may contribute to ASD pathogenesis [96, 129, 130]. Biological timing is critically important for neurodevelopment and brain plasticity [131]. Conceivably, the disturbances in the circadian system can lead to sleep disturbance, dysregulation of clock gene expression and diurnal oscillations of many hormones. All these can have detrimental consequences to neurodevelopment and lead to the emergence of a plethora of neurodevelopmental conditions including ASD (Fig. 4). Indeed, impairments of circadian rhythm for a few days can have an impact on the specialization and maturation of brain functions at certain times of development [132]. A study by Kobayashi et al. provides evidence that circadian clock genes *Clock* and *Bmal1* can control the timing of brain plasticity in the development of the neocortex and that the visual cortex of a *Clock* deficient mice shows delayed maturation of inhibitory PV cells [133]. Another study by Goto et al. reported a role for the clock gene *Nr1d1* in mouse brain development and possible implication in ASD [112]. Three *NR1D1* mutations are identified in ASD patients. Using mouse models, they further find that *Nr1d1* plays a pivotal role in cortical neuron migration, axon extension and dendritic arbor formation. Together these results suggest that functional defects in *Nr1d1* may be related to ASD pathophysiology [112]. More experimental evidence supporting a potential role of circadian disruption in the development of ASD is discussed in 4.4. Nevertheless, the mechanistic associations of ASD and circadian dysfunctions remain to be fully understood. Some of the other well-studied pathways in ASD, including the altered excitatory (glutamatergic)/inhibitory (GABAergic) pathways, oxidative stress, changes in the synaptic plasticity, and other metabolic pathways, may also serve as links between circadian dysfunction and ASD pathogenesis [134–137], as these pathways are directly or indirectly controlled by the circadian clock. Thus, it is possible that disruptions in the circadian clock may be responsible for the alterations of these pathways.

### The mTOR signaling pathway and ASD

The mTOR signaling cascade regulates numerous neuronal processes from proliferation, differentiation to plasticity and aging [14]. Dysregulation of the mTOR signaling pathway is implicated in the pathogenesis of various neurological disorders including ASD [14]. Hyperactivation of the mTOR pathway have been identified in several syndrome related to ASD [138]. For example, a study by Tang et al. demonstrated the link between ASD and mTOR where an increase in the dendritic spine density with reduced developmental spine pruning in layer V pyramidal neurons was observed in the temporal lobes of postmortem ASD samples [139]. The overactive mTOR signaling may also produce excessive spines thus correlating ASD and mTOR. mTORC1 regulates protein synthesis through downstream effector proteins 4E-BPs and S6Ks [11]. One possible explanation is that mTORC1 activation suppresses 4E-BPs and leads to dysregulated cap-dependent mRNA translation [140]. The complex includes eIF4E (the cap-binding protein), eIF4A (the RNA helicase), and eIF4G (the scaffolding protein bridging RNA to ribosome). The causal role of eIF4E in ASD was first pointed out by the discovery of individuals with ASD whose *EIf4e* gene had an activating mutation in its promoter region [141]. The development of ASD-like phenotypes via abnormal 4E-BP/eIF4E axis has been demonstrated in mouse models [142]. Dysregulated translation control may lead to abnormal expression of specific ASD risk genes [142, 143]. On the contrary, however, Nicolini et al. found decreased mTOR activities in the postmortem fusiform gyrus samples of patients with idiopathic autism [144]. Interestingly, Rosina et al. observed that there was an increased activities of mTOR and MAPK pathways in the peripheral blood samples of ASD patients [145].

Some of the strongest evidence that mTOR may also be required developmentally comes from genetic mutations associated with neurodevelopmental disorders. Mutations in negative regulators of mTORC1, such as *TSC1*, *TSC2* and *PTEN*, are found in monogenic ASD [14]. The frequent incidence of autism in the monogenetic mTORopathies has advocated a critical role for mTOR in the pathogenesis of autism [138]. The association of abnormal mTOR activities (particularly hyperactivation) with different syndromic forms of ASDs, such as TSC, Fragile X syndrome (FXS), Angelman syndrome, Hamartoma tumor syndrome, and Rett syndrome, has been documented in various clinical studies. Numerous animal studies reported that the deletion of various genes such as *Tsc1/2, Pten, Nf1, Fmr1* resulted in ASD-like phenotypes through the disruption of the mTORC1 mediated signaling. Firstly, studies show that ASD is strongly associated to TSC and the heterozygous deletion of *Tsc1* or *Tsc2* considerably increase the individual’s risk for ASD development [146]. Patients with mutations of *Tsc1* or *Tsc2*, upstream repressors of mTOR activities, exhibit an array of autistic features that resemble idiopathic autism [147]. The subjects with TSC have reported to show a 100-fold increase in the susceptibility of getting diagnosed with autism in comparison to the normal individuals. Further, the alterations in the TSC-related cell signaling also have a substantial role in ASD pathogenesis [146]. TSC1 or TSC2 normally form an mTOR inhibitor complex and thus their loss causes activation of mTOR signaling which in turn leads to mTORC1-dependent increase in the phosphorylation of S6, S6Ks, and 4E-BPs [146, 148, 149]. This reduced inhibition further causes the mTOR hyperactivity, altered protein synthesis, enhancement of proliferation. Also, a study by Alsaqati et al. demonstrated the TSC disorder which they characterized by hyperactivation of the mTORC1 pathway using the iPSC from ASD patients [150]. Secondly, the mutations in the *Fmr1* gene leads to FXS and associated to abnormalities in the mTOR-dependent protein synthesis. Also, the overactivation of mTORC1 has been reported to be associated with FXS [151]. In addition, a study by Hoeffer et al. reported an increase in the phosphorylation of mTOR, S6K1, AKT in their protein lysates in subjects with fragile X syndrome [151]. Dysregulation of mTOR signaling has been found in Fragile X Syndrome. A study by Sharma et al. provides a functional link between elevated mTOR signaling and aberrant synaptic plasticity in fragile X mouse. A study by Qin et al. illustrated that FMRP limits the protein synthesis via activation of metabotropic glutamate protein activation and the functional loss of this FMRP has reported to cause increased protein synthesis [152]. Further, dysfunctions of intracellular signaling pathways including mTOR and PTEN pathways are considered pathogenic in ASD [153]. In addition to this, about 1–5% of ASD cases have demonstrated *PTEN* gene mutations, a repressor of the phosphatidylinositol 3-kinase/Akt/mTOR signaling pathway. This result suggests that disinhibited mTOR signaling can be a contributing factor for ASD phenotypes in these cases. Moreover, the functional loss of *Pten* has also been reported to be linked to ASD. The *PTEN* gene mutations have been found in about 5% of ASD patients with macroencephaly [154]. As the role of PTEN in synaptic functioning is associated with ASD, this link can contribute to better understanding of the ASD pathology and promote potential new therapies. Further, 15% patients with neurofibromatosis type 1, a prototypical disorder caused by heterozygous mutations in the *Nf1* gene, meet the criteria for ASD [138, 155]. NF1 is a negative regulator of RAS and thus involved in the mTOR signaling regulation which further shows the link of ASD to mTOR signaling [156]. These findings found that mTOR signaling is dysregulated in different subset of ASD patients may help explain the wide degree of clinical severity and may help provide insight into the treatment strategies for treating individuals with ASD.

In addition to this, dysregulation of the mTOR pathway has also been implicated in animal models of ASD. A study by Nicolini et al. reported decreased mTOR signaling pathway in rats exposed to valproic acid indicated by a decrease in the phosphorylated and total mTOR, Akt, and 4E-BP1 and phosphorylated S6 protein in the VPA exposed rats [144]. Another study by Tang G et al. reported postnatal spine pruning defects, blockade of autophagy, and ASD-like social behaviors in the *Tsc2*^+/−^ ASD mice model suggesting that mTOR-regulated autophagy is required for developmental spine pruning [139]. Further, specific deletion of *Tsc2* in cerebellar Purkinje cells leads to autistic-like changes in mice, indicating cerebellar-specific mTOR signaling regulates mouse social behavior [157]. A study by Kotajima-Murakami et al. demonstrated the implication of over-activation of mTOR in the pathogenesis of syndromic ASD, such as TSC [148]. The results of this study showed that treatment with mTOR inhibitor rapamycin improved social interaction deficits in mouse models of TSC [148]. Enhanced mTORC1/S6K1 activities are found in a mouse model of FXS, the *Fmr1* knockout (KO) mice [158]. Moreover, a study by Yan et al. demonstrated that enhanced activity of mTORC1 was related to the reduction in the autophagy and protein degradation in *Fmr1* KO mice [159]. A study by Gantois et al. showed that hyperactivation of mTORC1 and ERK MAPK pathways is found in *Fmr1* knockout mice and metformin ameliorates core behavioral deficits by normalizing ERK hyperactivation [160]. Targeted *Pten* deletion in the forebrain leads to aberrant social behaviors in animals [140, 154]. Studies found that the *Pten* mutant mice showing deficits in the social behaviors resembling ASD individuals was associated with the hyperactivity of mTORC1 and its downstream pathway element S6K1. The studies also showed that rapamycin treatment reversed the ASD phenotypes in the *Pten* mutant mice further confirming the role of mTORC1 in ASD [161, 162]. Another study showed that treatment with rapamycin ameliorated the social deficits in the BTBR mouse model of ASD [163]. Further studies revealed that the *Eif4ebp2* KO and *Eif4e* overexpression generated an autistic-like behavioral manifestation, impaired social approach, and repetitive behaviors in mice [142, 143]. Thus, this evidence suggests a common biochemical link between dysregulated mTOR signaling and ASD.

### The crosstalk between mTOR and circadian clock and its implications in the development of ASD

Emerging evidence suggest that the mTOR pathway may serve as a link between circadian dysfunction and ASD pathogenesis. As mTOR activities in the brain are rhythmically regulated by the circadian clock and exhibit daily oscillations, dysfunction of the circadian clock can lead to aberrant temporal activities of the mTOR signaling and impair neurodevelopment [16]. Indeed, Fang et al. examined the effects of chronic disruption of circadian rhythms on neurodevelopment and animal behaviors in wild-type mice [6]. They find that aberrant light-dark cycle disrupts rhythmic clock gene expression in different brain regions including SCN and the hippocampus and leads to hyperactivation of mTORC1 and MAPK pathways in the brain. Adult WT mice raised in the aberrant light-dark cycle exhibit autistic-like behavioral changes, including impaired social interaction, communication, and repetitive behaviors. Genome-wide changes in gene expression are identified in the hippocampus. A number of ASD risk genes are differentially expressed, including *Pon1, Magel2, Ppp1r1b, Slc29a4, Ttc25, Dydc2, Fam92b, Ttn, Tcf7l2, Rorα, Foxp2, Rims3*, and *Satb2*. Aberrant synaptic transmission, immature dendritic spine morphology are also found in the hippocampus of these mice [6]. These results demonstrate that disruption of circadian rhythms during neurodevelopment can lead to aberrant mTOR activities and autistic-like molecular and behavioral changes in adult mice. In the future, it would be important to identify the developmental stages crucial to autism when the crosstalk between mTOR and the clock is most consequential. mTOR activities are dysregulated in monogenic mouse models of ASD, in which circadian dysfunctions are also identified. A study by Sawicka et al. demonstrated circadian rhythm defects in hippocampus dependent memory in the *Fmr1* knockout mice [164]. Moreover, Lipton et al. demonstrated that mutation of *Tsc1 or 2*, the mTOR repressor genes, leads to elevated protein levels of Bmal1, which in turn disrupts the circadian rhythms in mice [77]. It is not clear though in which developmental stage the disruption of clock function emerges and whether the clock dysfunctions contribute to the pathogenesis in these conical ASD models.

Recent evidence demonstrates that disruption of clock gene expression in specific brain regions can deregulate mTORC1 pathway, which in turn may lead to autism-like phenotypes in mice. The essential clock gene *Bmal1* is associated with human sociability and its missense mutation has been identified in ASD. A recent study by Liu et al. provides evidence that *Bmal1* disruption may contribute to the development of ASD-like traits in mice by mTORC1 overactivation [165]. They find significant social impairments, excessive stereotyped and repetitive behaviors, as well as motor learning disabilities in the *Bmal1* KO mice, all of which resemble core behavioral deficits in ASD. Similar autism-like behavioral changes phenotypes are also found in *Bmal1*^+/−^ mice [166]. Furthermore, pathological, and electrophysiological changes are found in the cerebellar Purkinje cells (PCs) of in the *Bmal1* KO mice. By ribosome profiling several signaling pathways of translational control, including mTORC1 signaling, are found to be dysregulated in the cerebellum of *Bmal1* KO mice. Interestingly, the antidiabetic drug metformin specifically reversed mTORC1 hyperactivation and alleviated major behavioral and PC deficits in *Bmal1* KO mice, suggesting mTORC1 hyperactivation may underlie these changes. Conditional *Bmal1* deletion only in cerebellar PCs can recapitulate autistic-like behavioral and cellular changes similar to those identified in *Bmal1* KO mice, suggesting that *Bmal1* disruption in the cerebellar PCs is responsible for changes in the *Bmal1* KO mice. Although it is found that *Bmal1* disruption in the cerebellar PCs reduces firing rates in these cells and therefore affects the output signals from the cerebellar cortex, it remains unclear how the *Bmal1* disruption in the PCs can affect the activities of the cerebello-thalamo-cortical circuit, which is thought to play a significant role in the pathogenesis of ASD [167]. Together, these results demonstrate a link between molecular clock dysfunction and ASD pathogenesis by mTORC1 hyperactivation.

## Circadian dysfunction, mTOR, and MD

### Introduction of MD

MD refer to conditions that severely affect the mood and its associated functions. According to DSM-5, MD broadly include major depressive disorders (MDD), bipolar disorder (BD) I and II, disruptive mood dysregulation disorder, persistent depressive disorder, cyclothymic disorder, and premenstrual psychotic disorder. MDD refers to a heterogeneous and multifactorial psychiatric illness acting at different levels such as psychological, biological, genetic, and social [168]. MDD is one of the most common mental disorders in the United States with a prevalence of about 7.8% of adults having at least one episode of mood disorders (2019; NIMH). The diagnosis of MDD includes consistent depressed/low mood, constant feeling of anhedonia, lack of concentration, poor sleep patterns, a state of worthlessness, changes in appetite, psychomotor impairments, and even suicidal thoughts. The prevalence of MDD is higher in females (9.6%) than in males (6%) [169, 170]. Also, it is maximum in the age group 18–25 (15.2%) has the highest MDD episodes [170]. BD is a serious type of MD identified by recurrent episodes of depression altering with periods of mania that are usually separated by periods of relatively normal mood and functioning [171].

Although a plethora of studies have been conducted to apprehend the causal mechanisms of MD, a clear outlook on the underlying mechanisms involved has not yet been possible. Nevertheless, MD are thought to be a multicausal disorder, the etiology of which is a combination of genetic, neurobiological, and environmental factors. The management of MD includes varied approaches such as pharmacological treatment options, psychotherapeutic interventional as well as lifestyle modifications. The combination of psychotherapy and medication has proved to be more effective than any of these treatments alone. The FDA-approved drugs for mood disorders treatment include SSRIs, SNRIs, Serotonin modulators, atypical antidepressants, and TCAs, MAOIs. Despite the presence of multiple treatment options for this disorder, a considerable population of affected individuals shows very low to no improvement in the symptoms. A major drawback of the present treatment options available is the failure of these drugs to target the complex pathophysiology of mood disorders without causing major side effects. The clinical use of ketamine (esketamine), a nasal spray for mood disorders is also limited due to its accompanying side effects (dissociative effects, change in sensory perception), and its potential abuse liability. Therefore, identifying novel molecular mechanisms and potential drug targets for the treatment of MD are urgently needed. Recent work has found significant associations between the circadian clock, mTOR signaling and MDs in human and animal studies.

### Clock dysfunction in MD

MD are often accompanied by disturbances or perturbations of the regular and daily circadian rhythms [172]. One of the most noticed disruptions occurs in the sleep-wake cycles of mood disorders patients [173–175]. For instance, patients with depression find difficulties in the initiation and maintaining their sleep. Further, there is shortening of the REM (rapid eye movement) latency and early awakening in the morning [172]. As the endogenous circadian system controls the daily physiological rhythms, this chronometry programming is often compromised in depressed people. In addition, the depressed patients often present abnormal levels and patterns of melatonin secretion, which in turn has an impact on the circadian controls [176, 177]. Also, agomelatine, a melatonergic agonist, was found to have therapeutic benefits in the patients with mood disorders [178, 179]. Therefore, the altered rhythms could be a possible biomarker for the diagnosis of mood disorders, developing therapeutic options, and further targeting the circadian system to improve mood.

A number of studies have identified disrupted clock gene expression in patients with MD. A study by Li et al. established a direct link between disruptions in the circadian patterns and MDD by developing a time-of-death analysis to 24 h sinusoidal gene expression data from postmortem brains of 34 MDD patients compared to controls [180]. This study indicates that the disruption of circadian gene expression (*BMAL1, BHLHE, PER1/2/3, BHLHE41*, and *NR1D1)* is linked to the functional regulation of various neuronal processes as well as behaviors (including mood) [180]. A study by Soria et al. found significant associations in *CRY1* (rs2287161), *NPAS2* (rs11123857), and *VIPR2* (rs885861) genes with patients with mood disorders (MDD and BD) [181]. Similarly, Lavebratt et al. studied the relation of genetic variability in the circadian clock linking genes in the predisposition to MDDs and found the association of the clock gene *CRY2* with depression [182]. Also, a study by Kovanen et al. demonstrated the association of *CRY2* genetic variants in the Finnish population with mood disorders, thus suggesting *CRY2* as the diagnostic marker for MDD [183]. Further, a study by Bruney et al. on the postmortem brain tissue of MDD patients showed dysregulated patterns of clock genes in different brain regions with the most robust changes in anterior cingulate (ACC) [184]. A study by Li et al. reported disruptions in the relative expression of clock genes mRNA (*PER1, PER2, CRY1, BMAL1, NPAS2*, and *GSK3β*) in patients with MDD as compared to healthy controls [185]. In addition, a study by Saus et al. indicated that abnormal processing of pre-miR-182 in patients carrying the T allele of the rs76481776 polymorphism may be a contributing factor to the disruptions in circadian rhythms in MDD patients with insomnia [186]. A cross-sectional study by Gouin et al. demonstrated the presence of higher mRNA levels of *Clock, Bmal1, Per1* in the patients with a history of depression as compared to the control or the non-depressed patients [187]. A genetic connection between circadian gene variation and major depression was again established by Shi et al., where genetic polymorphisms in circadian genes (especially *CLOCK* and *PER3*) were found to have an influence risk on developing depression in a sex- and stress-dependent manner [188]. Numerous studies show the association of genetic polymorphism in the circadian genes with MD populations [189–193]. Pirovano et al. found the association of MD and sleep disturbances with two new SNPs which are known downstream for T3111C polymorphism [194]. Some studies linking the disruption of various clock gene expression to human patients and animal models of MD are summarized in the table below (Table 1).

**Table 1:** Disruption of clock genes in mood disorders.

Human diseases or animal models	Involved genes	Main findings	References
MDD:592, Control:776	*ARNTL, CLOCK, NPAS2, PER3*	Mood disorders associates with a *CLOCK* variant in males and a *PER3* variant in females.	Shi et al. [242]
MDD:775, Control:440	*CRY1 NPAS2*	Significant association *CRY1* (rs2287161), *NPAS2* (rs11123857) and *VIPR2* (rs885861) genes in mood disorders samples.	Soria et al. [243]
MDD:34, Control:55	*BMAL1, PER1,2,3, NR1D1, DBP, BHLHE40, BHLHE41*	Time-of-death analysis to gene expression data of post-mortem brain of mood disorders patients showed association of canonical clock genes *BMAL1*(*ARNTL*), *PER*1-2-3, *NR1D1*(REV-ERBa), DBP, *BHLHE40* (DEC1), and *BHLHE41*(DEC2).	Li et al. [180]
MDD:12, Control:12	*PER1, PER2, PER3, CRY1, BMAL1, NPAS2, GSK-3β*	mood disorders patients showed disruptions in diurnal rhythms of *PERIOD1, PERIOD2, CRY1, BMAL1, NPAS2, GSK-3b* and disruptions in diurnal rhythms of melatonin, VIP, cortisol, ACTH, IGF-1release.	Li et al. [185]
MDD: 105, Control: 485	CRY1, CRY2, TEF	*CRY1* rs2287161 and *TEF* rs738499 are associated with the susceptibility of mood disorders. Carriers of *CRY1* rs2287161 were 1.91 times and *TEF* rs738499 were 2.66 times more likely to suffer from mood disorders	Hua et al. [190]
MDD:383, Control: 4154	*CRY2, PRKCDBP*	Association of *CRY2* (p<0.05) and novel associations of PRKCDBP rs1488864 with depressive disorders (p=0.02) and with major depressive disorder in specific (p=0.007).	Kovanen et al. [183]
BD: 215, Control: 773	*CSNK1E*	Association of SNP rs2075984 and bipolar disorder in both allelic (p= 0.003) and genotypic (p=0.006).	Lee at al. [244]
MDD: 359, Control: 341	*CLOCK*	Abnormal processing of pre-miR-182 in patients with T allele of rs76481776 polymorphism leads to dysregulated circadian rhythm in MDD patients with insomnia	Saus et al. [186]
BD:260, Control: 350	*CLOCK*	*CLOCK 3111T/C* showed significant allelic (*p* = 0.012) and genotypic (*p* = 0.033) associations with bipolar disorder.	Lee et al. [245]
BD: 121, Control: 121	*PERIOD*	*PER2* rs2304672 G allele frequency increased in risk for BD.	Yegin et al. [246]
Mouse model of depression and anxiety	*Bmal1*	Disruption of circadian rhythms by knocking down *Bmal1* expression in the SCN caused depression and anxiety-like behaviors in mice.	Landgraf et al. [207]
Mouse model of depression	*Cry1*	The *Cry1* knockout mice showed depressive-like behaviors.	Schnell et al. [208]
Rat model of depression	*Clock*	Sleep deprivation alters circadian oscillations of clock genes and causes depressive-like behavior in rats.	Xing et al. [209]
Rat model of depression	*Per1, Per2*, and *Bmal1*	*Per1, Per2*, and *Bmal1* is associated with the induction of a depression-like state in the chronic mild stress model in rats.	Christiansen et al. [210]
Mouse model of mania	*Clock*	*Clock* mutant mice show overall profile similar to human mania	Roybal et al. [200]
Mouse model of mania	*Clock*	Mice with a deletion of exon 19 in the *Clock* gene (ClockΔ19 mice) exhibit abnormal behaviors similar to BD mania.	van Enkhuizen et al. [247]
Mouse model of mania	*Clock*	Knockdown of *Clock* in ventral tegmental area of mice leads to mixed state of mania and depression like behavior	Mukherjee et al. [202]

**Fig. 5:**
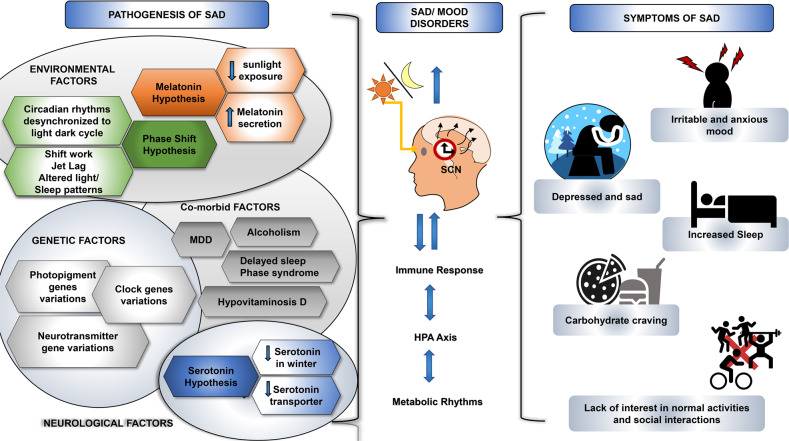
The mTOR signaling pathway may be the therapeutic target of multiple antidepressants. Exposure to stress or other negative stimuli may cause depression and related disorders by inhibition of the brain mTOR activities. On the contrary, anti-depressing agents or lifestyle may work through augmenting mTOR activities directly or indirectly in the brain.

Seasonal affective disorder (SAD) is a special form of MDD that exhibits a seasonal pattern. In SAD, the recurrent major depressive episodes are associated with shortened day length (photoperiod) and commonly occurs during autumn or winter. SAD is more prevalent in the places where the day length changes according to different seasons are extreme such as high latitudes. The circadian phase shifts and melatonin daily rhythms are considered to be a possible justification of this seasonal form of depression [195]. The demonstration of light during the night increases alertness and thus suppresses the release of melatonin in mammals. As per the circadian phase shift theory hypothesis, late sunrise in the winter seasons leads to a significant delay in the circadian rhythmicity [196]. A study by Partonen et al. reported variations in the circadian genes *PER2*, *ARNTLl*, *NPAS2* in patients with SAD [197]. Similarly, a study by Kim et al. reported that the polymorphisms of *CLOCK, NPAS2* and *ARNTL* are associated with the seasonal variations in behavior and mood. Further, a few studies show that the presentation of bright light early in the morning was helpful in the treatment of SAD possibly due to phase advancing the circadian system putting it back in sync with the sleep/wake cycle [198, 199]. There are other hypotheses such as serotonin hypothesis, genetic factors, and other comorbid conditions such as alcoholism, delayed sleep phase syndrome that can help to understand the etiology of SAD (Fig. 5).

The strong relationship between disrupted circadian rhythms and MD is demonstrated in mouse models. In a series of elegant studies, the McClung group have established an animal model of BD using the ClockΔ19 mutant mice and investigated the underlying neural mechanisms [200]. Mice carrying the ClockΔ19 mutation display behaviors that are similar to human mania, including rapid mood cycling, hyperactivity, decreased sleep, lowered depression-like behavior, lower anxiety, and an increase in reward seeking [201]. Knockdown of *Clock* in the ventral tegmental area results in a mixed state of mania and depression-like behavior [202]. Mechanistically, the abnormalities in VTA dopamine neuron firing and reduced level of cholecystokinin, and excitatory signaling via Gria1 expression in the NAc appears to underlie the manic-like phenotypes [203–205]. Valproate can be used to treat the manic and mixed phases of bipolar disorder. Using the ClockΔ19 mouse model, Logan et al. found that the therapeutic actions of VPA for bipolar mania is partially via the inhibition of histone deacetylase protein 2 in the VTA [206]. Landgraf et al. demonstrated that the disruption of circadian rhythms by knocking down *Bmal1* expression in the SCN caused depression and anxiety-like behaviors in mice [207]. A study by Schnell et al. reported that the *Cry1* knockout mice showed depressive-like behaviors [208]. Xing et al. reported that sleep deprivation alters circadian oscillations of clock genes and causes depressive-like behavior in rats [209]. Similarly, a study by Christiansen et al. reported that altered expression of clock genes (*Per1, Per2*, and *Bmal1*) is associated with the induction of a depression-like state in the chronic mild stress model in rats [210]. A study by Guo et al. showed that Abelson helper integration site 1 (*Ahi1*) deficient mice exhibit depressive-like behaviors via changes in the circadian clock pathways [211]. A study by LeGates et al. reported that aberrant light directly impairs mood through intrinsically photosensitive retinal ganglion cells. The antidepressant fluoxetine ameliorates the depressive-like behavior in mice under irregular light cycles by modulating the increased corticosterone levels, suggesting that this mechanism is associated with depression caused by jet lag [212].

### mTOR and MD

**Fig. 6:**
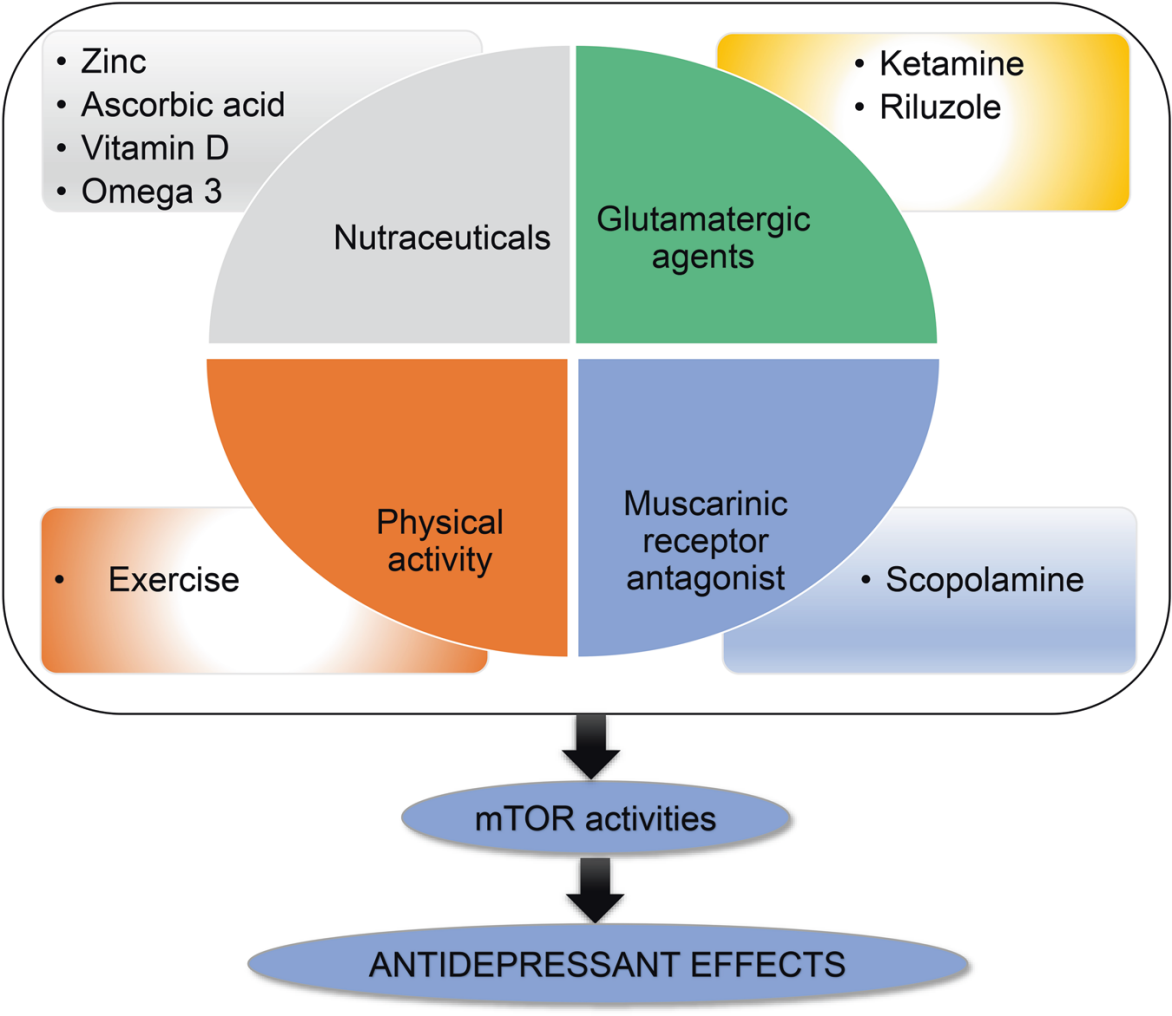
Seasonal Affective Disorder: Pathogenesis, symptoms, and circadian regulation. The various hypothesis such as phase shift, melatonin, and serotonin hypothesis, have been proposed for pathogenies of SAD. These factors may convergently influence the brain circadian clock, which may further influence the neural mechanisms underlying SAD and other mood disorders. Like mood disorders, SAD is also characterized by poor mood, anhedonia, loss of energy, weight gain, hypersomnia etc.

Within the last decade, the glutamatergic system has been implicated in the neurobiology and treatment of depression. Also, the NMDA, N-methyl-D-aspartate receptor antagonists have appeared as the central players in the pathophysiology as well as treatment of depression. Ketamine, an NMDA receptor antagonist have been a successful drug for the different forms of treatment-resistant depression [213]. However, the regular clinical use of ketamine in patients with depression is restricted due to its accompanying harmful effects such as changes in the sensory perception, dissociative properties, and its abuse liability. These limitations have engaged the scientists in exploring the primary targets and thorough mechanism of action underlying the antidepressant effect of ketamine which can result in novel therapeutics for depression. These new treatment options are anticipated to mimic ketamine’s unique antidepressant actions while lacking its undesirable effects. Several reports over the years have linked the mTOR signaling pathway to the antidepressant effect of ketamine. A role of mTOR in the anti-depressive effects of ketamine has been explored in a pioneering study by Li et al., where it was found that ketamine activates the mTOR pathway and causes an increase in the levels of synaptic signaling proteins as well as increased number and functionality of dendritic spines in the prefrontal cortex of rats [214]. Particularly, the administration of ketamine is implicated in increased phosphorylation of mTOR, S6K1, and 4E-BPs. These chemical changes further were correlated with an increase in the levels of synaptic proteins, synapse number in dendritic spines in the PFC. Furthermore, studies have shown the abrogation of the ketamine’s anti-depressive effects on pre-administration with rapamycin which in turn suggests the involvement of mTOR signaling in the mechanism of action of ketamine [213–215]. Further, ketamine has also been observed to disinhibit translation of BDNF in a manner dependent on the S6K1 substrate eEF2K [216]. Therefore, mTOR is an emerging signaling pathway of interest in mood disorders pathophysiology and treatment (Fig. 6).

Repressed mTOR activities are found in patients with MDs. A study by Jernigan et al. reported a decrease in the expression of mTOR and its downstream components (S6K, eIF4E, and eIF4B) in the postmortem samples of the prefrontal cortex of 12 patients with MDs as compared to healthy controls [217]. A similar outcome regarding mTOR was also observed in the blood samples from BD patients taken during the depressive episodes where expression of *mTor, Akt* were reduced in comparison to the healthy participants. These peripheral alterations indirectly correspond to that observed in the brain samples [218]. In addition to this, the administration of subanesthetic ketamine in three patients with depression caused an abrupt decrease in the depressive symptoms which may be associated with an acute increase in the plasma mTOR, GSK-3 expression, and phosphorylation of the eEF2 [219]. In a study by Wang et al., bioinformatics analysis was performed to investigate the mechanism of MDs in males and found that downregulated *microRNA-124-3p* suppresses the mTOR signaling pathway by targeting DNA damage-inducible transcript 4 (DDIT4) in the male population with mood disorders, thus suggesting miRNA as a gender-specific novel target for MDs [220]. A study by Zhu et al. reported genome-wide profiling of DNA methylation and gene expression in monozygotic twin pairs with mood disorders and found distinct modulation enriched in signaling pathways such as insulin receptor signaling, growth factor receptor signaling, and mTOR signaling [221]. A study by Nowak et al. revealed the subanesthetic dose of ketamine shows an antidepressant effect by stimulating the mTOR-associated gene expression [222]. In addition, an open-label study on treatment-resistant depression patients by Roy et al. reported ketamine infusion improved the brain atrophy as well as increased the insulin/mTOR /GSK3 β in the responders [223].

Inhibition of the mTOR pathway can lead to changes in affective behaviors in animals, whereas enhancing mTOR activity can be therapeutic to some models of mood disorders. A recent study by Koehl M et al. found that inhibition of the mTOR signaling pathway via deletion of *S6K1* leads to anxiety-like behaviors in mice [224]. Mice and rats exposed to chronic unpredictable stress (CUS) exhibited depressive-like behaviors associated with a reduction in phosphorylation levels of mTOR and its downstream signaling components, such as S6K, in the prefrontal cortex [225], hippocampus [226], and amygdala [227]. An in vitro and in vivo study by Harraz et al. reported that the antidepressant effect of ketamine via mTOR is mediated by inhibition of nitrergic Rheb degradation [228]. A study by Szewczyk et al. found that the antidepressant action of zinc is via activation of mTOR-dependent signaling pathway [229]. Further, a study by Gordillo-Salas et al. reported that the antidepressant effect of GluN2A receptor antagonist, NVP-AAM077 occurred via increased GluA1 subunit of AMPA and mTOR signaling [230]. Similarly, other studies have explored the antidepressant effects of various drugs and found activation of the mTOR signaling as a common pathway [231, 232]. Overall, mTOR signaling mediates the behavioral response of many antidepressant drugs [57, 214, 216, 233–236], which also indicates a critical role of the mTOR signaling in depression.

Little is known, however, regarding the role of mTOR as a potential link between circadian disruption and the pathogenesis of mood disorders. It is well established that photoperiod regulates affective behaviors in diurnal and nocturnal animals [237]. Evans et al. found that the day length profoundly regulates the rhythms of clock gene expression and neuronal network properties in the SCN and presumably also in other brain regions that exhibit circadian rhythms of gene expression [238, 239]. Interestingly, circadian mTOR activities in the SCN are also regulated by photoperiod [16, 240]. As the SCN is the master circadian pacemaker that regulates rhythms of gene expression and neuronal activities in other brain regions, it is expected that photoperiod will also regulate mTOR rhythms in other brain regions that are important to regulate affective behaviors such as the prefrontal cortex (PFC). Along these lines, genetic repression of mTORC1/S6K1 activities in PFC can lead to depressive-like behaviors whereas increase expression of S6K1 can produce antidepressant effects in rats [241]. Together, these results suggest a model in which photoperiod may regulate animal affective behaviors by regulating rhythmic mTOR activities in specific brain regions.

## Conclusions

Circadian rhythm is ubiquitous, and rhythmic gene expression is found in a variety of brain regions. Thus, numerous neurophysiological processes exhibit significant daily oscillations in activities and functions. Intact circadian rhythm is critical for mental health, as disruption of daily rhythms by either environmental or genetic factors can lead to or exacerbate neurological and psychiatric problems in laboratory and clinical studies. Indeed, disruption of clock gene expression and circadian rhythm is frequently found in common psychiatric diseases such as ASD and MD. mTOR signaling is a fundamentally important signal transduction pathway, the disruption of which has been implicated in ASD and MD. As mTOR activities are closely regulated by the circadian clock and functionally integrated with the circadian timing process, circadian dysfunction inevitably leads to deregulation of temporal mTOR activities in the brain, which in turn may contribute to pathophysiological changes associated with psychiatric disorders. Although the underlying mechanisms remain to be fully understood, the interactions between the circadian clock, mTOR signaling, and psychiatric disorders appear to be significant in ASD and MD and should be considered in basic research and pharmaceutical developments. As mTOR inhibitors are FDA approved drugs, understanding the interactions between the circadian clock, mTOR, and psychiatric disorders may open new therapeutic avenues to regulate the brain clock function and treat psychiatric diseases.
